# A novel prognostic cancer-related lncRNA signature in papillary renal cell carcinoma

**DOI:** 10.1186/s12935-021-02247-6

**Published:** 2021-10-18

**Authors:** Binghai Chen, Di Dong, Qin Yao, Yuanzhang Zou, Wei Hu

**Affiliations:** 1 Department of Urology, Affiliated Hospital of Jiangsu University, Zhenjiang, Jiangsu China; 2 Department of Andrology, The First Affiliated Hospital of University of South China, Hengyang, Hunan China

**Keywords:** Papillary renal cell carcinoma, TCGA, LncRNA, Prognosis, RP11-63A11.1

## Abstract

**Background:**

Papillary renal cell carcinoma (pRCC) ranks second in renal cell carcinoma and the prognosis of pRCC remains poor. Here, we aimed to screen and identify a novel prognostic cancer-related lncRNA signature in pRCC.

**Methods:**

The RNA-seq profile and clinical feature of pRCC cases were downloaded from TCGA database. Significant cancer-related lncRNAs were obtained from the Immlnc database. Differentially expressed cancer-related lncRNAs (DECRLs) in pRCC were screened for further analysis. Cox regression report was implemented to identify prognostic cancer-related lncRNAs and establish a prognostic risk model, and ROC curve analysis was used to evaluate its precision. The correlation between RP11-63A11.1 and clinical characteristics was further analyzed. Finally, the expression level and role of RP11-63A11.1 were studied in vitro.

**Results:**

A total of 367 DECRLs were finally screened and 26 prognostic cancer-related lncRNAs were identified. Among them, ten lncRNAs (RP11-573D15.8, LINC01317, RNF144A-AS1, TFAP2A-AS1, LINC00702, GAS6-AS1, RP11-400K9.4, LUCAT1, RP11-63A11.1, and RP11-156L14.1) were independently associated with prognosis of pRCC. These ten lncRNAs were incorporated into a prognostic risk model. In accordance with the median value of the riskscore, pRCC cases were separated into high and low risk groups. Survival analysis indicated that there was a significant difference on overall survival (OS) rate between the two groups. The area under curve (AUC) in different years indicated that the model was of high efficiency in prognosis prediction. RP11-63A11.1 was mainly expressed in renal tissues and it correlated with the tumor stage, T, M, N classifications, OS, PFS, and DSS of pRCC patients. Consistent with the expression in pRCC tissue samples, RP11-63A11.1 was also down-regulated in pRCC cells. More importantly, up-regulation of RP11-63A11.1 attenuated cell survival and induced apoptosis.

**Conclusions:**

Ten cancer-related lncRNAs were incorporated into a powerful model for prognosis evaluation. RP11-63A11.1 functioned as a cancer suppressor in pRCC and it might be a potential therapeutic target for treating pRCC.

**Supplementary Information:**

The online version contains supplementary material available at 10.1186/s12935-021-02247-6.

## Background

Renal cell carcinoma (RCC) is a main human malignancy threatening people’s health globally. It is mainly divided into three catalogues, including clear cell RCC (ccRCC), papillary RCC (pRCC) and chromophobe RCC (chRCC), among which pRCC accounts of the second type with 15–20% of cases [1, 2]. PRCC is further divided into two subtypes, but the classification remains unsatisfactory [3]. Neither radiotherapy nor chemotherapy is effective in pRCC. In addition, targeted therapy that widely used in ccRCC is not efficient in pRCC either [3, 4]. Therefore, it is urgent to explore promising biomarker and therapeutic target to improve pRCC patients’ prognosis.

In recent decades, long non-coding RNAs (lncRNAs) are reported to be aberrantly expressed in many malignancies and involved in the oncogenesis and progression of human cancers, and they have great potential to serve as biomarkers for cancer diagnosis and targets for cancer treatment [5]. LncRNAs play roles in multiple cellular processes of cancer, including cell survival, migration, invasion, metastasis, and epithelial–mesenchymal transition (EMT), etc. [6, 7]. Additionally, lncRNAs are crucial regulators in many aspects, such as target mRNA expression regulation and cancer immunity regulation [8, 9]. What’s more, a series of lncRNAs have been identified as cancer-related lncRNAs [10–13]. For more cancer-related lncRNAs identification, different methods were performed and corresponding database was established [14, 15]. Therefore, lncRNAs, especially cancer-related lncRNAs are promising biomolecules in human cancers.

Immlnc database is a web-based resource to investigate the immune-related function of lncRNAs across cancer types [16]. Moreover, it provides a larger number of cancer-related lncRNAs in seventeen cancer types. In this study, we aimed to combine the Immlnc and The Cancer Genome Altas (TCGA) databases to identify prognostic cancer-related lncRNAs and construct a risk model for prognosis prediction in pRCC. As expected, we finally identified 26 prognostic cancer-related lncRNAs and constructed a prognostic risk model with ten lncRNAs (including RP11-573D15.8, LINC01317, RNF144A-AS1, TFAP2A-AS1, LINC00702, GAS6-AS1, RP11-400K9.4, LUCAT1, RP11-63A11.1, RP11-156L14.1).

More interestingly, RP11-63A11.1 was a renal tissue-specific lncRNA among the ten lncRNAs. It was reported that RP11-63A11.1 was significantly down-regulated in pRCC samples and correlated with clinicopathological features of pRCC patients [17, 18], which was consistent with the analysis of ours. However, the role of RP11-63A11.1 is still not completely understood in pRCC. Thus, we further detected the expression of RP11-63A11.1 in pRCC cells and investigated its role on cell survival and apoptosis, which would add more convincing evidence to previous conclusion.

## Methods

### Acquisition and processing of samples data

The KIRP RNA-seq data (TCGA-KIRP) were acquired from UCSC, and clinical data of pRCC was downloaded from TCGA database. A total of 289 tumor samples and 32 matched non-tumor samples were included based on The Cancer Genome Atlas (TCGA) database. The samples with overall survival (OS) less than 1 month were eliminated, since these patients were likely to die of other reasons, such as serious infection [19, 20]. A total of 275 pRCC samples were screened out and divided into training set with 136 cases and testing set with 139 cases by the “caret” package of R software at random. The testing set and the combination set (the 275 samples) were used to validate the outcomes acquired from the training set.

### Identification of differentially expressed mRNAs and cancer-related lncRNAs

The differentially expressed mRNAs (DEMs) between pRCC and its corresponding normal samples were analyzed by the “limma” package of R software. The significant cancer-related lncRNAs in pRCC were obtained from the Immlnc database. And then, their expression data in TCGA database was extracted and the “limma” package of R software was performed to analyze the differentially expressed cancer-related lncRNAs (DECRLs). |log2FC| > 2 and adjust P value < 0.01 were considered as the threshold. The DECRLs were removed if their expression values were equal to 0 in more than 135 samples. Finally, 367 DECRLs were screened out for further investigation.

### Identification of prognostic cancer-related lncRNAs and establishment of a signature for prognosis prediction

We firstly implemented the univariate Cox regression analysis on the 367 DECRLs and clinical survival data in training set to determine prognostic cancer-related lncRNAs. And then we further analyzed the significant results using multivariate Cox regression analysis to generate a risk model. Each patient obtained a risk score with this formula: riskscore = β1 × exp(lnc1) + β2 × exp(lnc2)+···+βi × exp(lnci). Explnc is the expression value of DECRLs in the patients, while β refers to the regression co-efficient of cancer-related lncRNAs generated from the risk model in training set. Based on the medial riskscore, patients in all three sets were divided into high and low risk groups. Subsequently, the survival rate of the two groups was evaluated. The area under the ROC curve (AUC) was utilized to assess the precision of the risk model. The testing set and combination set were utilized to validate the above results.

### Independence analysis of the cancer-related lncRNA signature in pRCC

Both the univariate and multivariate Cox regression were implemented to analyze the independence of this cancer-related lncRNA signature from the clinical features of pRCC patients, including age, gender, tumor stage and T classification. The data of M and N classifications were excluded in the independence analysis because the number of samples with undetermined classification was far beyond half of the all samples.

### Weighted gene co-expression analysis (WGCNA) and gene enrichment analysis

In order to explore correlation between lncRNAs signature and biologic functions of pRCC, we performed WGCNA to construct the gene co-expression modules among differentially expressed mRNAs as previous study mentioned [21]. The modules with the maximal absolute module significance correlated to lncRNAs signature were screened out. Then, gene enrichment analysis of genes in the most lncRNAs signature related module was carried out using DAVID [22]. Gene Ontology (GO) terms with false discovery rate (FDR) less than 0.05 were considered as significant biologic functions.

### Aberrant expressed mRNAs correlated with RP11-63A11.1

To explore the potential mechanism of RP11-63A11.1 involved in the progression of pRCC, we analyzed the aberrant expressed mRNAs which were correlated with RP11-63A11.1 expression. We listed aberrant expressed mRNAs in the combination set using Pearson method. The positively related mRNAs were screened out with correlation coefficient over 0.3.

### Prediction of subcellular location

As the function of lncRNA is associated with its subcellular localization, the sequence of the RP11-63A11.1 was obtained from the LNCipedia website (https://lncipedia.org/) [23]. The obtained sequence was input into the lncLocator website to predict the subcellular localization of the lncRNA [24]. The results were obtained as a score for each potential subcellular location for RP11-63A11.1, including the cytoplasm, nucleus, ribosome, cytosol and exosome.

### The association of RP11-63A11.1 expression and clinicopathological characteristics and prognostic indicators of pRCC

Among the ten lncRNAs, RP11-63A11.1 was primarily expressed in renal tissues and it was the most significant one with the lowest P value for OS in training set. Therefore, we further analyzed the association of RP11-63A11.1 expression and clinicopathological features (including gender, age, tumor stage and TNM classifications) as well as prognostic indicators of pRCC. Furthermore, we also investigated the expression and functions of RP11-63A11.1 in vitro.

### Cell culture

Human pRCC SK-RC-39 cell line and normal renal cell line HK-2 were purchased from Cellcook Biotech (Guangzhou, China). The cell mediums (including RPM1-DMEM and RPM1-1640) and Fetal Bovine Serum (FBS) were purchased from Fcmacs Biotech (Nanjing, China). Antibiotic mixture (penicillin and streptomycin) and l-glutamine were purchased from Sangon Biotech (Shanghai, China). 57 mL FBS and 5.7 mL antibiotic mixture as well as 5.7 mL l-glutamine were added into cell mediums to generate complete mediums. SK-RC-39 and HK-2 were fed in complete 1640 and DMEM medium, respectively. Both the two kinds of cell lines were cultured in the 5% CO_2_ incubator at 37 °C.

### RP11-63A11.1 over-expression

Lenti-sgRNA RP11-63A11.1 was constructed by lenti-sgRNA. We also generate the SK-RC-39^dCas9+MPH+^ cells before they were infected with lenti-sgRNA RP11-63A11.1. We then used the puromycin to screen the cells and picked up clones. The over-expressions of RP11-63A11.1 were subsequently validated in clones. The clones with elevated RP11-63A11.1 were used for further assays.

### RT-qPCR assay

Following the manufacturer’s protocol, EASYspin Tissue/Cell RNA Rapid Extraction Kit (Chaoyan Biotech, Shanghai, China) were used to extract the total RNA of cells. Then reverse transcription kit (Vazyme, Nanjing, China) was used to synthesize cDNA. Based on the instruction of ChamQ SYBR qPCR Master Mix Kit (Vazyme), qPCR amplification was performed on QuantStudio 5. GAPDH was considered as the internal reference. The expression level of RP11-63A11.1 was analyzed by 2^−ΔΔCt^ method. Primers: RP11-63A11.1, forward, 5′-TCAGCAGGGTTTAGAGCAGC-3′; reverse, 3′-CTGAGGTTTCCATGCTGCTG-5′; GAPDH, forward: 5′-GAGTCAACGGATTTGGTCGT-3′, reverse: 5′-TTGATTTTGGAGGGATCTCG-3′.

### Cell proliferation assay

CCK-8 (Vazyme, Nanjing, China) assay was applied for the evaluation of cell proliferation ability. In brief, after counting with the cell count plate, 1 × 10^4^ cells were seeded into each experimental well of a 96-well plate and then cultured at 37 °C for 24 h, 48 h and 72 h. After adding 10% CCK-8 reagent into the corresponding wells, the absorbance value at 450 nm was measured.

### Cell apoptosis assay

For cell apoptosis detection, we prepared and adjusted the cell concentration to 1 × 10^6^/mL. Then Annexin V-Alexa Fluor 488/PI Apoptosis detection kit (Fcmacs Biotech, Nanjing, China) was used for cell staining and flow cytometer was used for the assessment of cell apoptosis.

### Western Blot

The total protein was extracted using radioimmunoprecipitation assay solution (Beyotime, Shanghai, China) before polypropylene gel electrophoresis. Then the protein was further transferred to polyvinylidene difluoride membranes (Millipore, USA). After blocked by 3% bovine serum albumin (Fcmacs Biotech, Nanjing, China) in room termperature, the membrane was incubated with primary antibody overnight. Sources of primary antibodies (cleaved PARP, cleaved Caspase 3, Bcl-2, Bax, Cytochrome C and GAPDH) were from Cell Signaling (Danvers, MA, USA). RASSF10 antibody was from Abcam (Cambridge, England, UK). After the membrane was incubated with secondary antibody, the proteins were visualized by the gel-imaging system.

### Statistical analysis

R software 4.0.2 and SPSS 25 were used to implement statistical analysis in this study. Univariate and multivariate Cox regression analysis were applied to generate a cancer-related lncRNA signature and identify independent prognostic factors for pRCC. The survival rate of pRCC patients in two risk-groups was appraised by Kaplan–Meier method. The *P* value < 0.05 was generally considered statistically significant.

## Results

### Differentially expressed mRNAs and cancer-related lncRNAs in pRCC

**Fig. 1:**
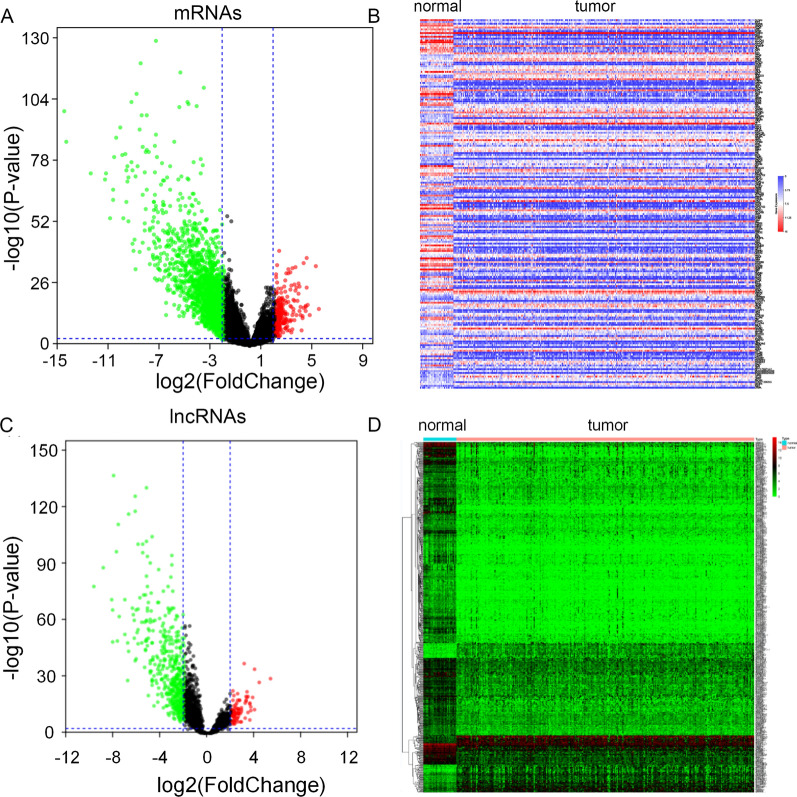
Differentially expressed mRNAs and cancer-related lncRNAs in pRCC. **A** Volcano plot of differentially expressed mRNAs. **B** Heatmap of the top 200 differentially expressed mRNAs. **C** Volcano plot of differentially expressed cancer-related lncRNAs. **D** Heatmap of differentially expressed cancer-associated lncRNAs. The red and green points represent up-regulation and down-regulation, respectively

A total of 1872 cancer-related lncRNAs with expression data in pRCC were extracted (Additional file 1: Table S1). Through the “limma” package of R software, 2030 DEMs were identified, including 219 up-regulated DEMs and 1811 down-regulated DEMs (Fig. 1A, B), while 579 differentially expressed cancer-related lncRNAs (DECRLs) were found, including 84 up-regulated DECRLs and 495 down-regulated DECRLs (Fig. 1C, D). The DECRLs were removed if their expression values were equal to 0 in more than 135 samples. 367 DECRLs were finally determined for further analysis.

### Identification and validation of cancer-related lncRNA signature

**Fig. 2:**
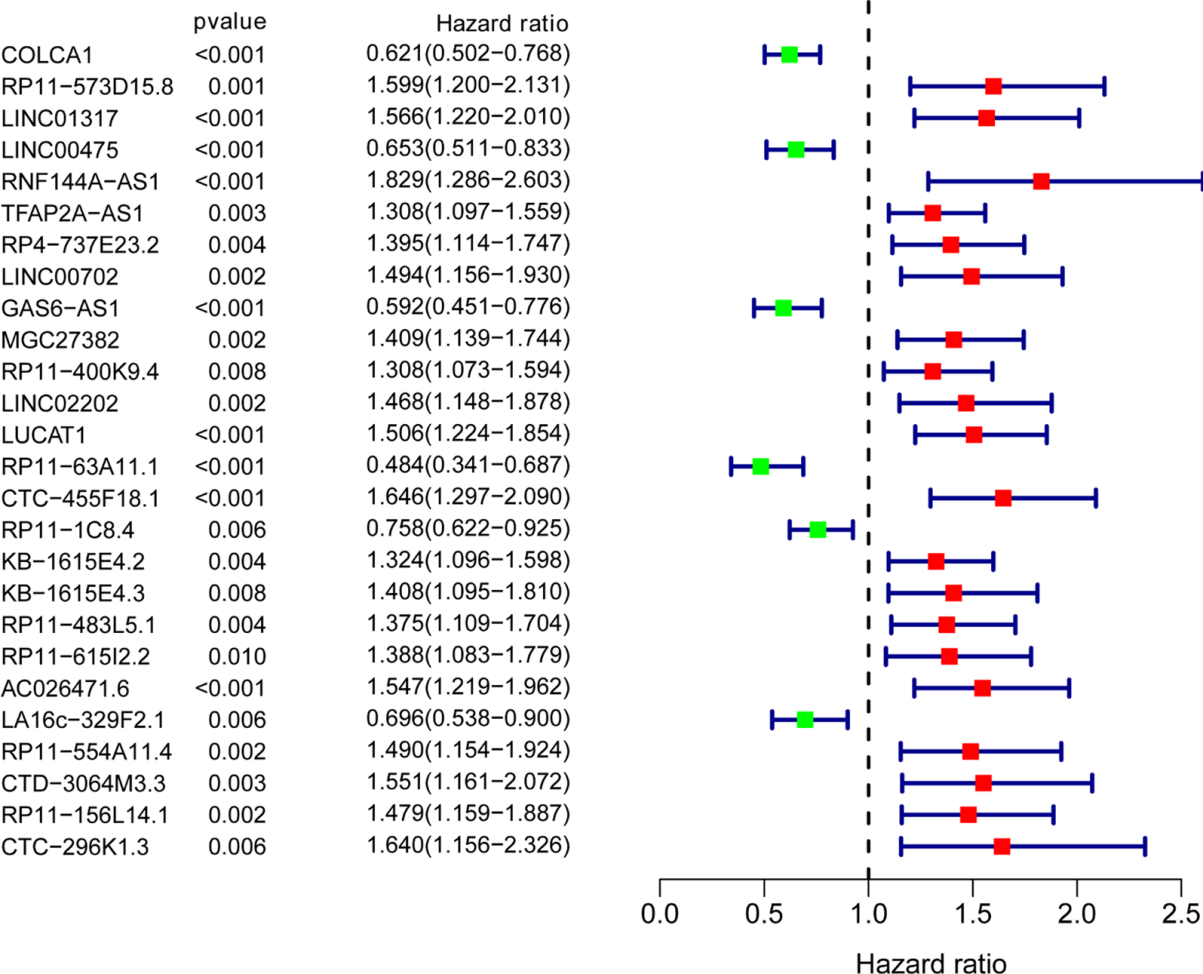
Forest plot of the prognostic cancer-related lncRNAs in pRCC. Hazard radio > 1 (red) means the lncRNAs are high risk genes and hazard radio < 1 (green) means the lncRNAs are low risk genes

**Fig. 3:**
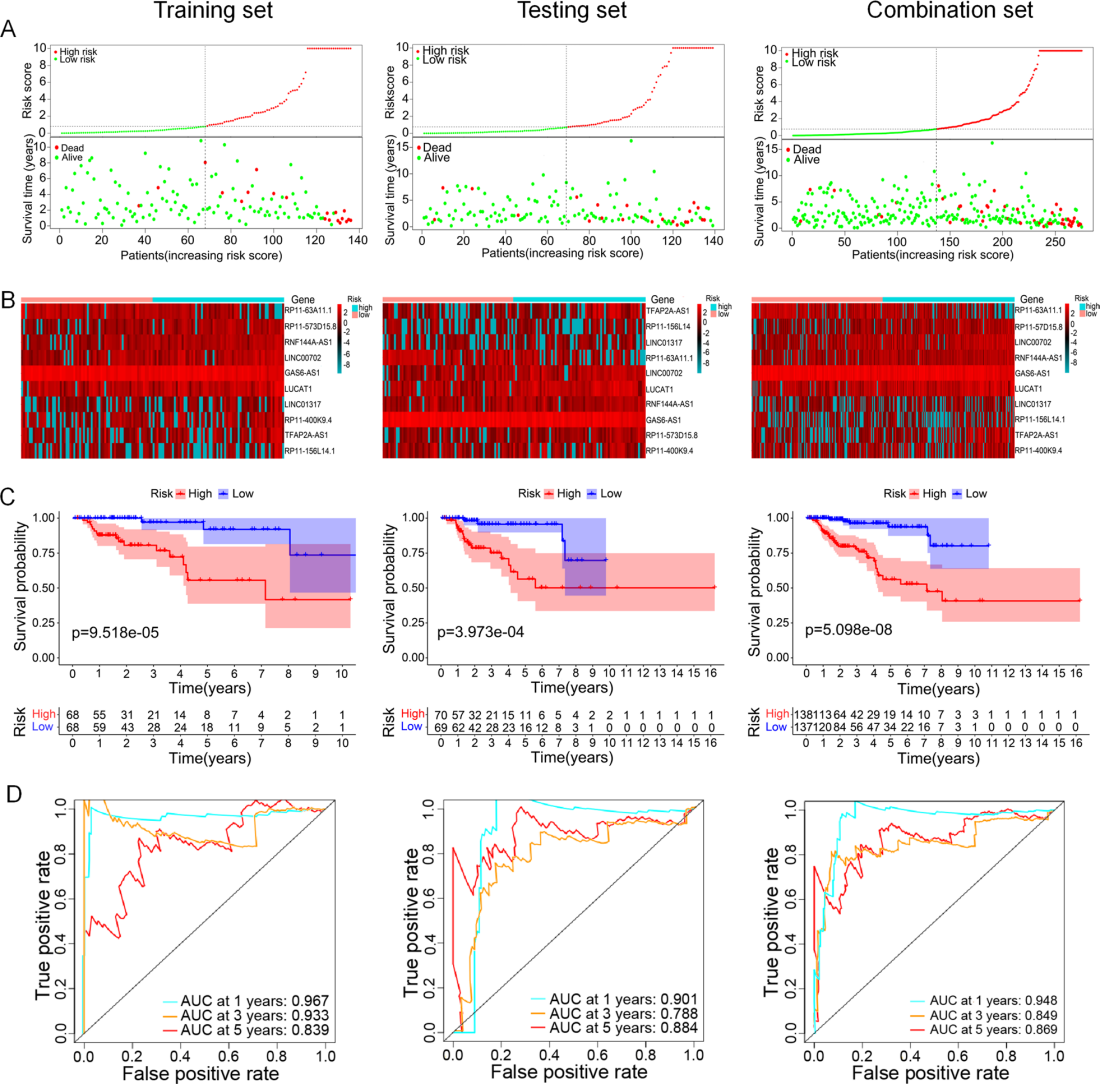
The cancer-related lncRNA signature in the three patients sets. **A** pRCC patients classified by risk score and their survival status. **B** Heatmap of the cancer-related lncRNA signature. **C** The survival curves of the cancer-related lncRNA signature. **D** The ROC curves of the cancer-related lncRNA signature at 1, 3, and 5 years

The expression profile of 367 cancer-related lncRNAs with OS data was analyzed by the univariate Cox regression to identify the prognostic lncRNAs in pRCC in the training set first. There were 26 lncRNAs identified as prognostic cancer-related lncRNAs in total (Fig. 2). Subsequently, multivariate Cox regression was conducted to generate a risk model of prognosis. 16 genes were screened off by the regression analysis. Then, the remaining 10 lncRNAs (RP11-573D15.8, LINC01317, RNF144A-AS1, TFAP2A-AS1, LINC00702, GAS6-AS1, RP11-400K9.4, LUCAT1, RP11-63A11.1, and RP11-156L14.1) were included in the model (Table 1). Riskscore = 0.7953 × exp(RP11-573D15.8) + 0.4074 × exp(LINC01317) + 0.5932 × exp(RNF144A-AS1) + 0.2636 × exp(TFAP2A-AS1) + 0.5327 × exp(LINC00702) − 0.6131 × exp(GAS6-AS1) − 0.8397 × exp(RP11-400K9.4) + 0.3506 × exp(LUCAT1) − 0.6670 × exp(RP11-63A11.1) − 0.6442 × exp(RP11-156L14.1). According to the riskscore formula, the riskscore of each pRCC patient in all three sets was calculated. On the basis of median riskscore value, pRCC cases were segmented into high and low risk groups. As shown in Fig. 3A, the mortality rate of pRCC patients was progressively increased with the rising of risk score in training-set, testing-set and combination-set. The levels of the ten lncRNAs in two groups were shown in Fig. 3B. The survival curves displayed that patients in high risk group had a shorter survival time than those in low risk group (Fig. 3C). The precision of the 10 cancer-related lncRNA signature in predicting prognosis of pRCC were analyzed through the ROC curves. The results demonstrated that all of the AUC values at 1, 3, and 5 years were over than 0.75 in the training-set, testing-set and the combination-set (Fig. 3D), indicating the ten cancer-related lncRNAs signature had a good sensitivity and specificity in prognosis prediction.

**Table 1:** Ten cancer-related lncRNAs used for model construction

Symbol	ID	HR	Low 95% CI	High 95% CI	P value	Coefficient
RP11-573D15.8	ENSG00000197099	2.2151	1.4438	3.3982	0.0003	0.7953
LINC01317	ENSG00000203386	1.5029	0.9733	2.3205	0.0661	0.4074
RNF144A-AS1	ENSG00000228203	1.8097	0.9352	3.5019	0.0782	0.5932
TFAP2A-AS1	ENSG00000229950	1.3016	0.9183	1.8449	0.1386	0.2636
LINC00702	ENSG00000233117	1.7035	0.9748	2.9767	0.0614	0.5327
GAS6-AS1	ENSG00000233695	0.5416	0.3708	0.7912	0.0015	− 0.6131
RP11-400K9.4	ENSG00000237807	0.4318	0.2353	0.7925	0.0067	− 0.8397
LUCAT1	ENSG00000248323	1.4199	1.0692	1.8857	0.0154	0.3506
RP11-63A11.1	ENSG00000250781	0.5133	0.3423	0.7695	0.0012	− 0.6670
RP11-156L14.1	ENSG00000265702	0.5251	0.3029	0.9104	0.0218	− 0.6442

### The cancer-related lncRNA signature was an independent factor in prognosis prediction

We further evaluated whether the identified cancer-related lncRNA signature was an independent factor for prognosis prediction in pRCC. Co-variables including age, gender, tumor stage and the risk model in training-set, testing-set and combination-set were analyzed by Cox regression analysis. The outcomes from univariate Cox regression revealed that tumor stage and the risk model were significantly relevant to OS in all three sets. Multivariate Cox regression analysis suggested that tumor stage and risk model were independent factors in the combination set. The results were listed in Table 2. Taken together, the cancer-related lncRNA signature was an independent factor for prognosis prediction in pRCC.

**Table 2:** Univariate and multivariate Cox regression of the cancer-related lncRNA signature and clinical features in predicting survival

Variables	Univariate Cox regression		Multivariate Cox regression	
	HR (95 % CI)	P value	HR (95 % CI)	P value
Training set				
Age	0.9822 (0.9438–1.0221)	0.3767	1.0038 (0.9630–1.0463)	0.8581
Gender	0.4531 (0.1660–1.2370)	0.1224	0.4317 (0.1436–1.2977)	0.1347
Tumor stage	3.1198 (1.8313–5.3147)	< 0.0001	2.4005 (0.9082–6.3452)	0.0774
T classification	2.2977 (1.3818–3.8204)	0.0013	0.8041 (0.3037–2.1291)	0.6608
riskScore	1.0084 (1.0054–1.0114)	<0.0001	1.0060 (1.0019–1.0102)	0.0038
Testing set				
Age	1.0232 (0.9823–1.0659)	0.2702	1.0172 (0.9672–1.0697)	0.5075
Gender	0.9556 (0.3160–2.8902)	0.9360	0.7268 (0.2087–2.5312)	0.6162
Tumor stage	2.2629 (1.5483–3.3074)	< 0.0001	1.6502 (0.7047–3.8642)	0.2486
T classification	2.1696 (1.4254–3.3023)	0.0003	1.5756 (0.5769–4.3033)	0.3751
riskScore	1.0119 (1.0070–1.0168)	< 0.0001	1.0126 (1.0057–1.0196)	0.0004
Combination set				
Age	1.0036 (0.9756–1.0324)	0.8033	1.0167 (0.9863–1.0479)	0.2847
Gender	0.6599 (0.3170–1.3734)	0.2663	0.7614 (0.3577–1.6208)	0.4795
Tumor stage	2.5702 (1.8967–3.4829)	< 0.0001	2.7575 (1.5812–4.8087)	0.0004
T classification	2.1839 (1.5849–3.0093)	< 0.0001	0.7615 (0.4087–1.4188)	0.3909
riskScore	1.0085 (1.0060–1.0110)	< 0.0001	1.0061 (1.0032–1.0090)	< 0.0001

### Stratification analysis

**Fig. 4:**
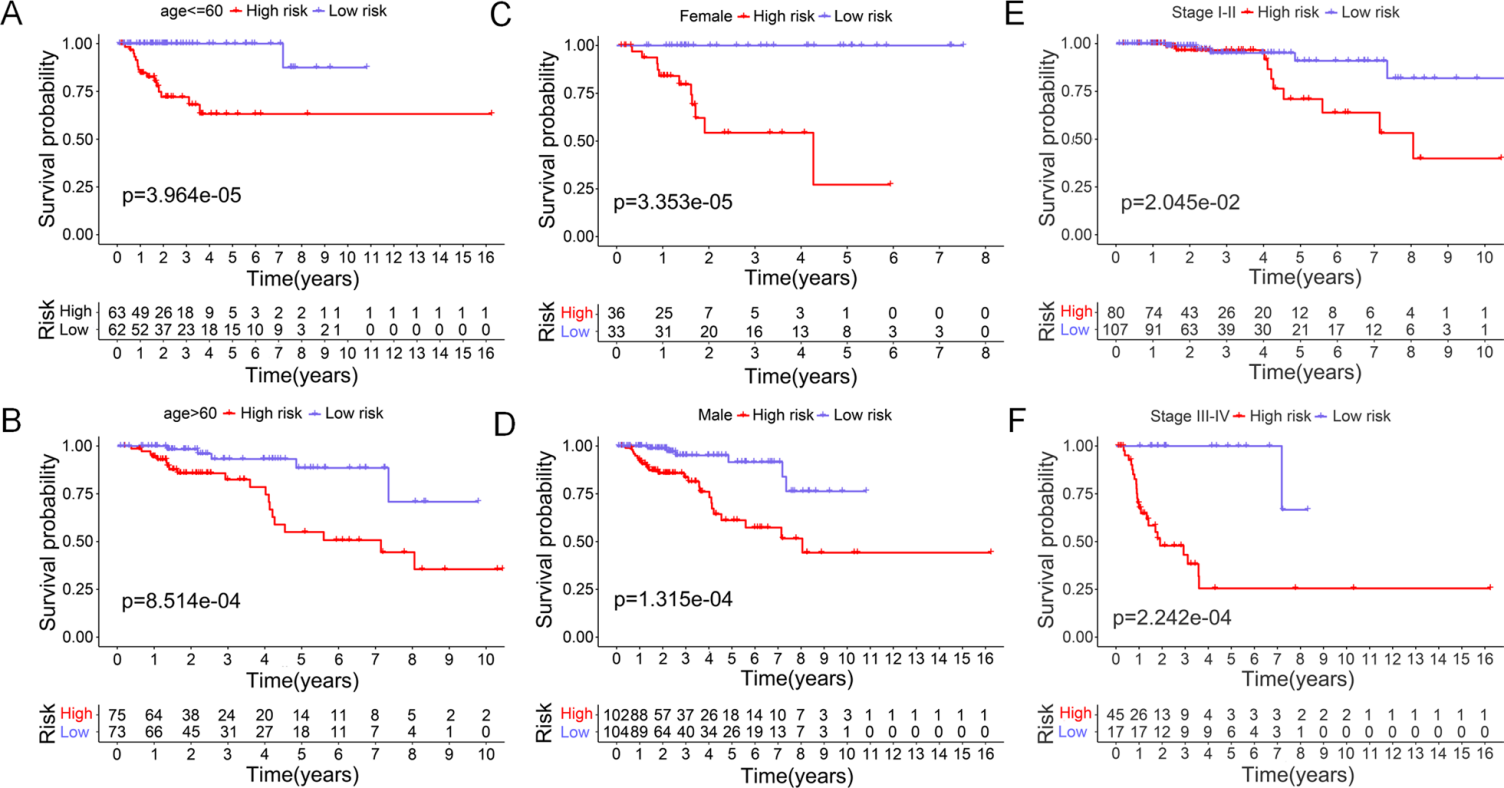
The survival curves of the cancer-related lncRNA signature in stratification of clinical features of pRCC patients. **A** Age ≤ 60 years, **B** age > 60 years, **C** female patients, **D** male patients, **E** stage I–II, **F** stage III–IV

By performing stratification analysis on the clinicopathological features of pRCC patients in the combination cohort, we revealed that the cancer-related lncRNA signature was a good predictor. As shown in Fig. 4A, B, both older (> 60 years) and younger (≤ 60 years) pRCC cases in high risk group exhibited a worse prognosis compared with those cases in low risk group. Of note, similar results of the cancer-related lncRNA signature were found in different genders (Fig. 4C, D) and tumor stages (Fig. 4E, F).

### The cancer-related lncRNA signature was associated with biological processes

**Fig. 5:**
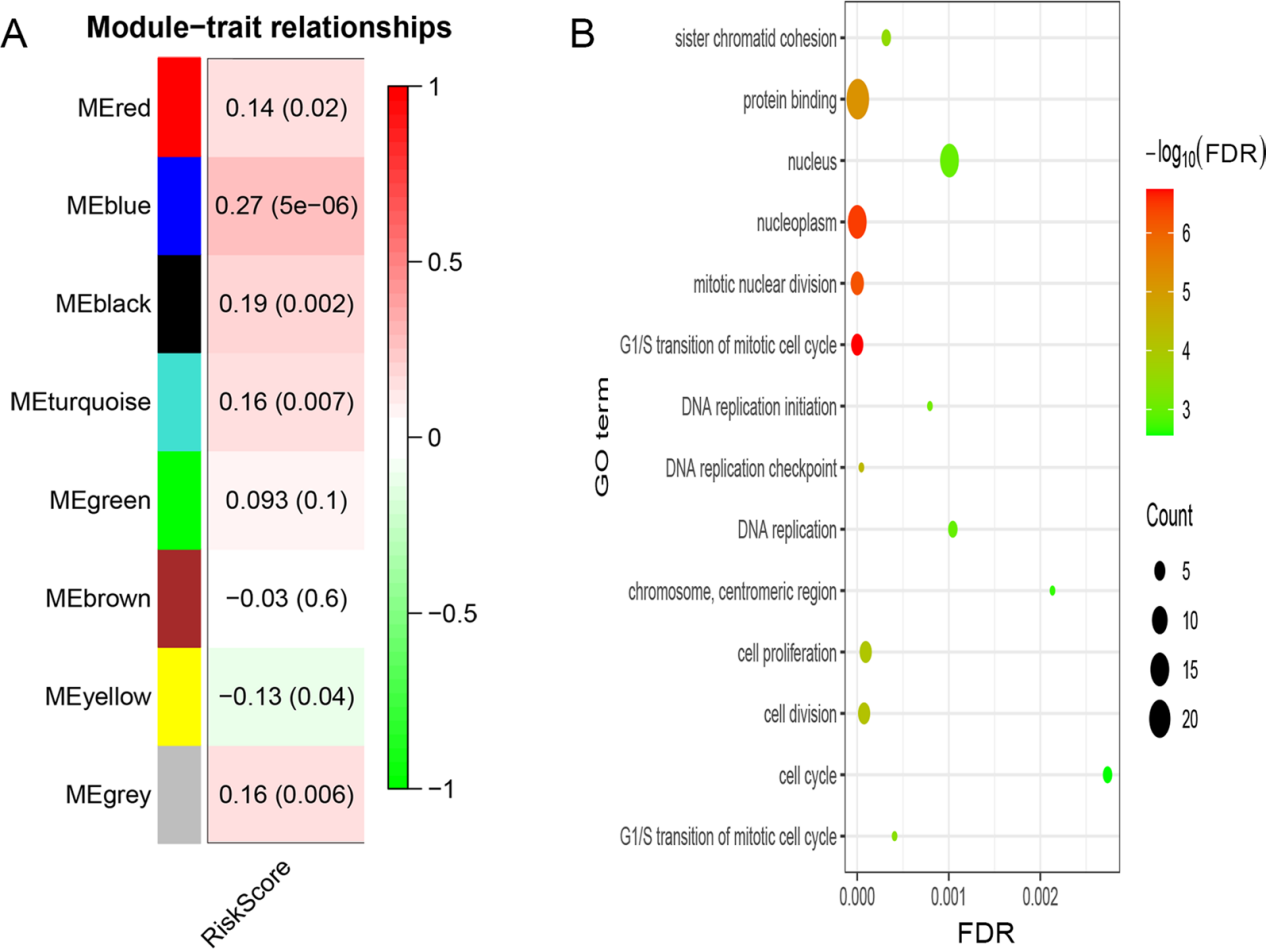
Weight gene co-expression analysis and function enrichment analysis. **A** The gene modules associated with the cancer-related lncRNA signature identified by weight gene co-expression analysis. **B** Gene oncology terms of significant gene modules associated with the cancer-related lncRNA signature

The cancer-related lncRNA signature was vital in prognosis of pRCC patients; therefore this signature should be closely associated with biological processes of pRCC. 2030 DEMs were used to construct 8 similar expression modules by average linkage clustering. The relevance with P value between each module and cancer-related lncRNA signature was listed in every module (Fig. 5A). The most negative related module (yellow module, R = − 0.13, P = 0.04) and positive related module (blue module, R = 0.27, P = 5e−06) were selected out for gene enrichment analysis. The FDR of GO term of genes in yellow module was over than 0.05. The genes in blue module were mainly involved in cell division, cell proliferation, and cell cycle process, such as G1/S transition of mitotic cell cycle, sister chromatid cohesion (Fig. 5B), etc.

### RP11-63A11.1 was associated with many clinical characteristics and prognostic indicators of pRCC

**Fig. 6:**
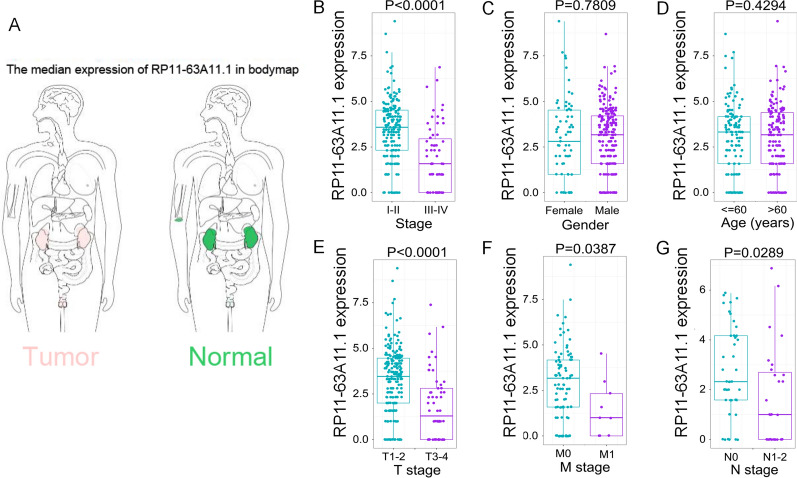
Expression of RP11-63A11.1 in different clinicopathological features. **A** Bodymap of RP11-63A11.1 in human normal tissues and tumor tissues. **B** RP11-63A11.1 expression in different tumor stage (I–II vs. III–IV). **C** RP11-63A11.1 expression in different gender (female vs. male). **D** RP11-63A11.1 expression in different age group. **E** RP11-63A11.1 expression in different T classification (T1–T2 vs. T3–T4). **F** RP11-63A11.1 expression in different M classification (M0 vs. M1). **G** RP11-63A11.1 expression in different N classification (N0 vs. N1–N2)

**Fig. 7:**
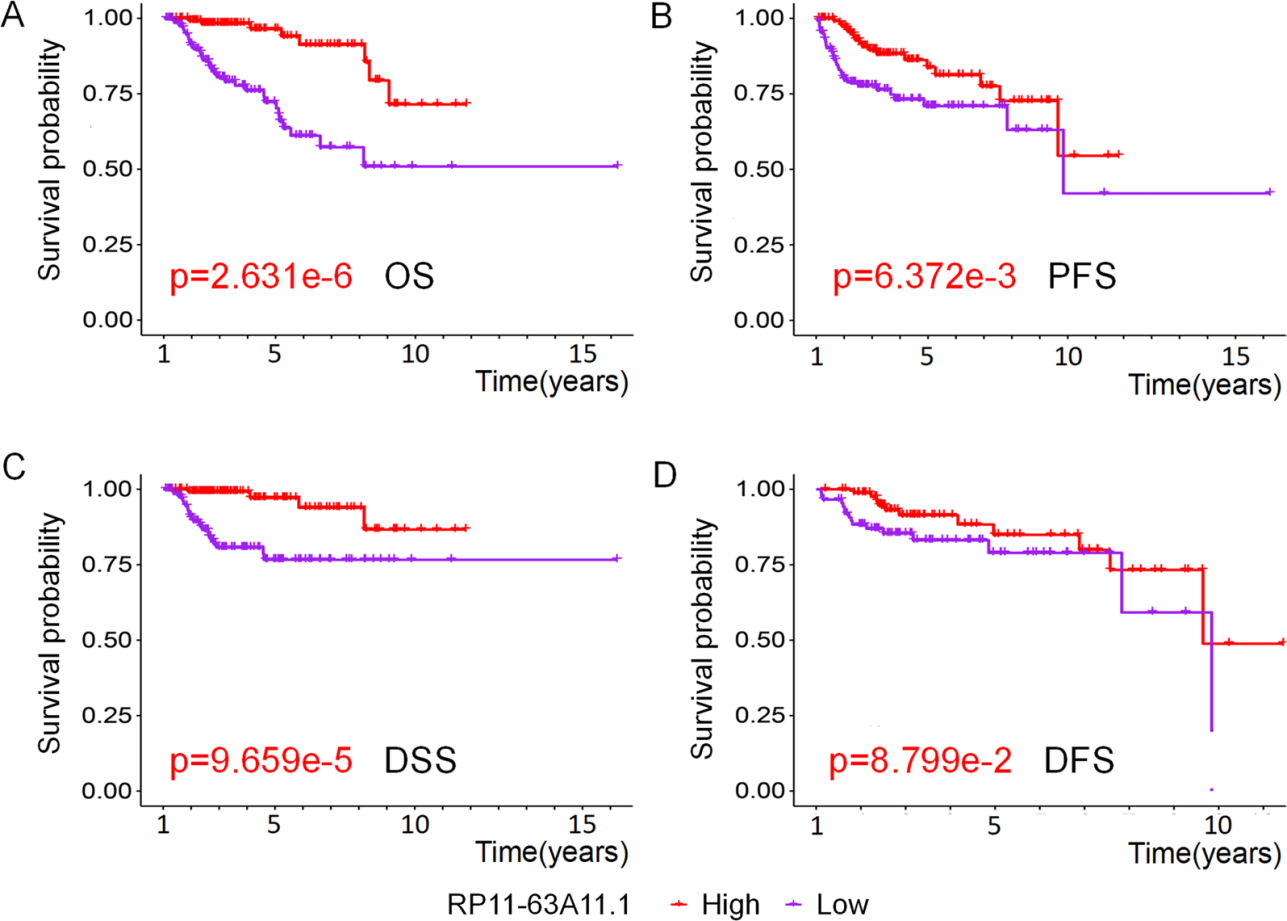
The association of RP11-63A11.1 expression with prognostic indicators in pRCC. **A** Higher level of RP11-63A11.1 indicates favorable OS. **B** Higher level of RP11-63A11.1 indicates favorable PFS. **C** Higher level of RP11-63A11.1 indicates favorable DSS. **D** There is no statistical difference of RP11-63A11.1 in DFS

Among all the 10 lncRNAs in the signature, RP11-63A11.1 was the most significant one with the lowest P value (P = 0.0014) by Kaplan-Meier method in the training set (Additional file 2: Fig. S1). In order to further understand the role of RP11-63A11.1 in pRCC, we analyzed the association of RP11-63A11.1 with the clinical features including gender, age, clinical stages, TNM classifications, OS, disease-free survival (DFS), disease-specific survival (DSS), progression-free survival (PFS). In the GEPIA database, we found that RP11-63A11.1 was mainly expressed in kidney, suggesting RP11-63A11.1 may be a renal tissue-specific lncRNA (Fig. 6A). It was indicated that the level of RP11-63A11.1 was significantly higher in lower tumor stages and TNM classifications, while it did not show significant difference in age and gender (Fig. 6B–G). Of note, higher level of RP11-63A11.1 indicates favorable OS (Fig. 7A), PFS (Fig. 7B), as well as DSS (Fig. 7C). Although there is no significant difference of DFS between high expression group and low expression group (Fig. 7D), RP11-63A11.1 could be a very critical prognosis predictor based on the data of OS, DSS, and PFS.

### Elevated RP11-63A11.1 inhibited survival and induced apoptosis of pRCC cells

**Fig. 8:**
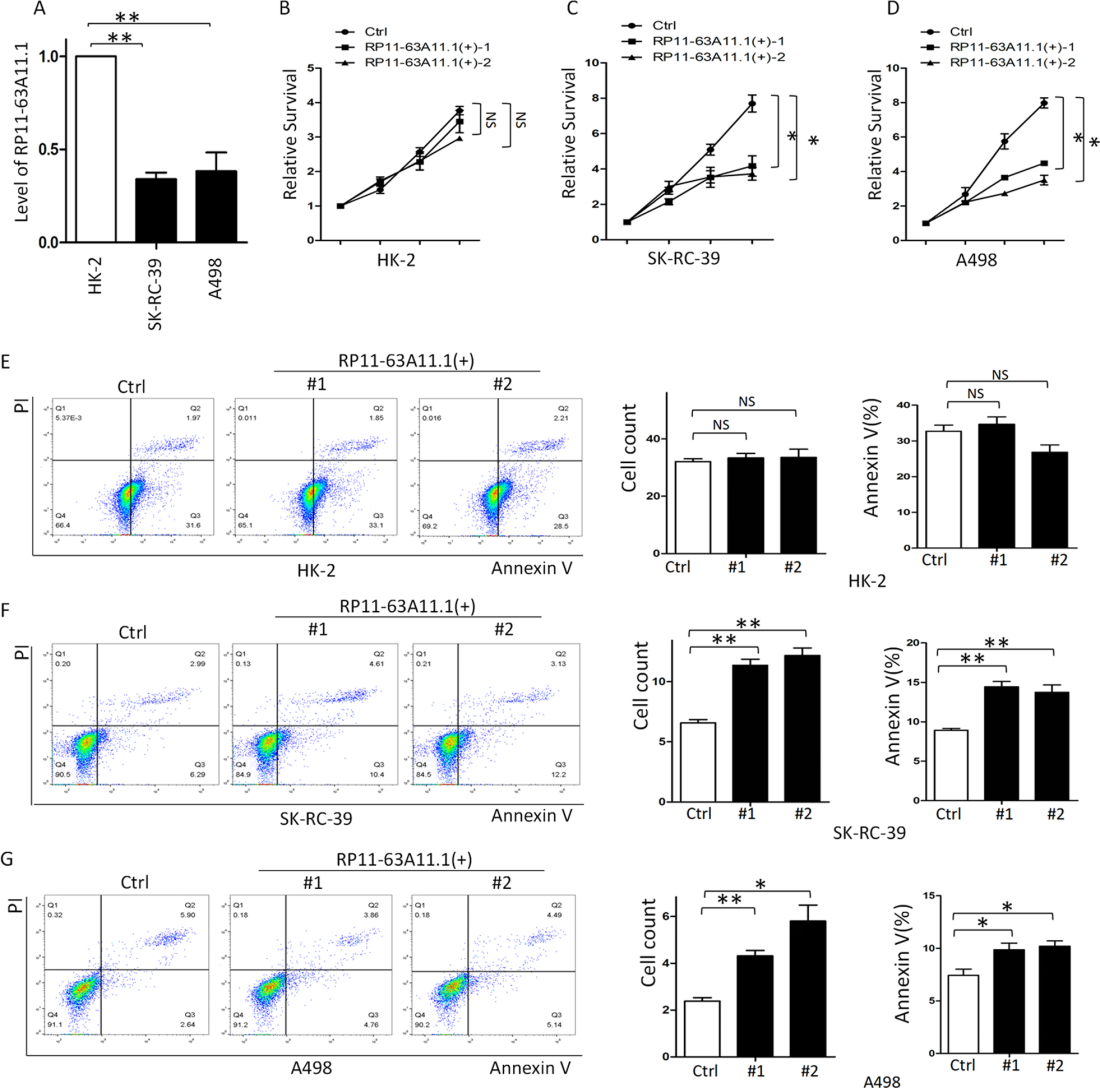
Elevated RP11-63A11.1 inhibited survival and induced apoptosis of pRCC cells. **A** RP11-63A11.1 expression was decreased in SK-RC-39 and A498 cells compared with that in HK-2. **B** Over-expressed RP11-63A11.1 didn’t inhibit the cell survival of HK-2. **C** Elevated RP11-63A11.1 significantly inhibited the cell survival of SK-RC-39 cells. **D** Elevated RP11-63A11.1 significantly inhibited the cell survival of A498 cells. **E** Over-expressed RP11-63A11.1 didn’t affect the apoptosis of HK-2. **F** Elevated RP11-63A11.1 significantly induced the cell apoptosis of SK-RC-39 cells. **G** Elevated RP11-63A11.1 significantly induced the cell apoptosis of A498 cells. *P < 0.05, **P < 0.01, NS: P > 0.05

**Fig. 9:**
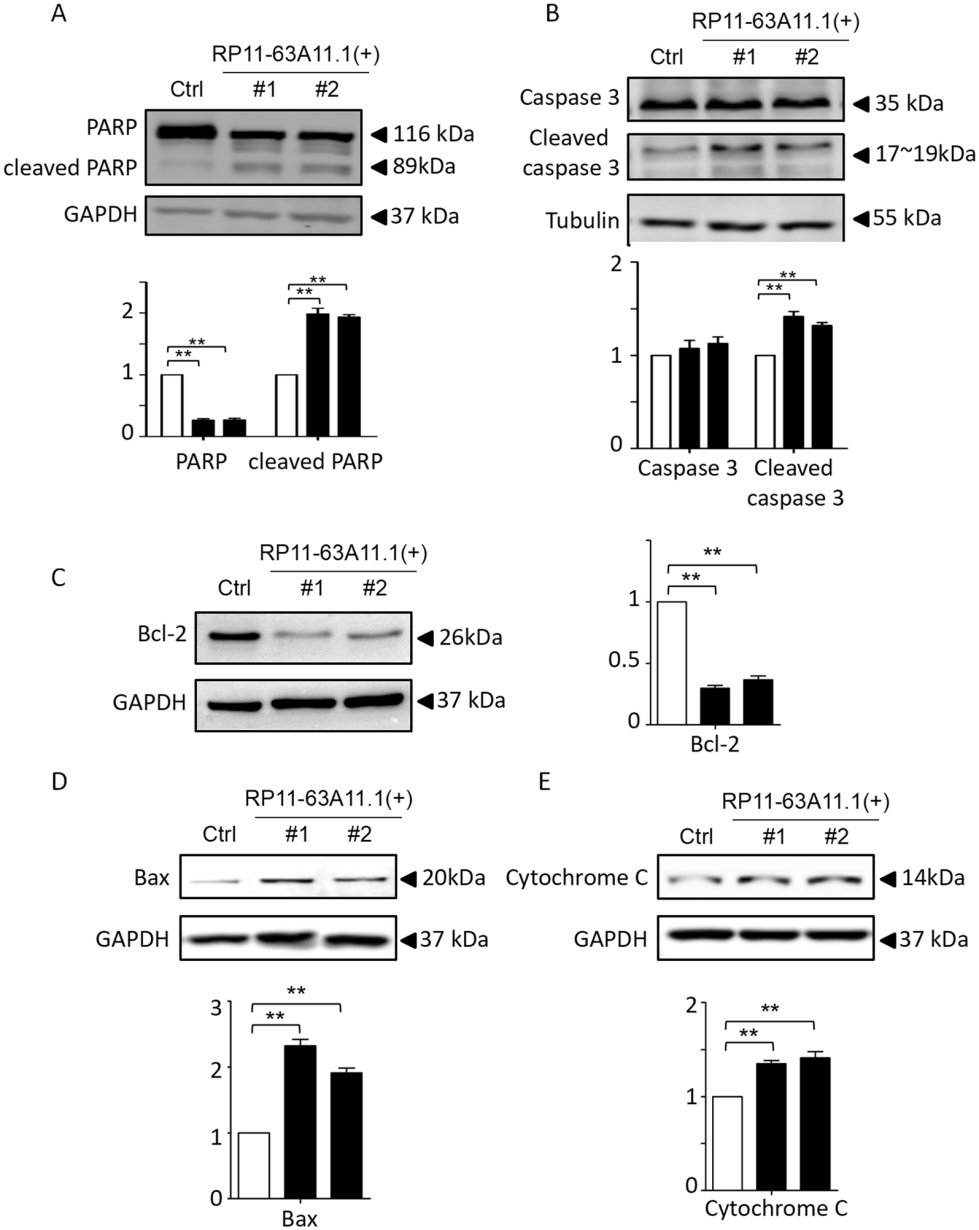
Elevated RP11-63A11.1 regulated five apoptotic markers. **A** Over-expression of RP11-63A11.1 increased level of cleaved-PARP while uncleaved-PARP significantly decreased. **B** The introduction of RP11-63A11.1 elevated cleaved-Caspase-3. **C** Over-expression of RP11-63A11.1 suppressed the expression of Bcl-2. Bax (**D**) and Cytochrome C (**E**) both increased upon introduction of RP11-63A11.1 *P < 0.05, **P < 0.01

In order to validate the role of RP11-63A11.1, we determined cell survival and apoptosis in pRCC cell line (SK-RC-39). We firstly determined the level of RP11-63A11.1 in pRCC cells. The result showed that the expression of RP11-63A11.1 was decreased in pRCC cells compared with normal renal cells (HK-2) (Fig. 8A). Then, we generated 2 clones with CRISPR/dCas9 system to elevate the level of RP11-63A11.1 in both SK-RC-39 and A498 (Additional file 3: Fig. S2). Subsequently, the cell survival assay indicated that elevated RP11-63A11.1 didn’t inhibit the HK-2 (Fig. 8B); however, it significantly inhibited the cell survival of SK-RC-39 and A498 (Fig. 8C, D). In consistent with that, the elevated RP11-63A11.1 didn’t affect the HK-2 (Fig. 8E) but induced the cell apoptosis of SK-RC-39 (Fig. 8F) and A498 (Fig. 8G), suggesting that RP11-63A11.1 could be a suppressive gene in pRCC. Over-expressed RP11-63A11.1 might be an effective way in therapy. Moreover, five apoptotic markers were investigated. It was shown in Fig. 9 that over-expression of RP11-63A11.1 increased level of cleaved-PARP while uncleaved-PARP significantly decreased (Fig. 9A). Similarly, we found the introduction of RP11-63A11.1 elevated cleaved-Caspase-3 (Fig. 9B), adding more evidence to the apoptosis induced by RP11-63A11.1. Moreover, over-expression of RP11-63A11.1 suppressed the expression of Bcl-2 (Fig. 9C), which was an inhibitor of apoptosis. On the other hand, Bax (Fig. 9D) and Cytochrome C (Fig. 9E) both increased upon introduction of RP11-63A11.1, which was consistent with those data in cell apoptosis assay.

### Analysis of the potential mechanism of RP11-63A11.1 in pRCC

How the RP11-63A11.1 regulates the apoptosis progression in pRCC cells is still unknown. To further investigate the possible mechanism, we first predict the subcellular location. The sequence information of RP11-63A11.1 was obtained from LNCipedia (Additional file 4: Table. S2). It is indicated in the LncLocator that RP11-63A11.1 is probably located in the cytoplasm (Table 3), suggesting that RP11-63A11.1 may serve as a ceRNA (competing endogenous RNA). Then, we showed all the genes which were positively correlated with RP11-63A11.1 (Additional file 5: Table S3). Among all the positively correlated genes, we found that RASSF10 was attractive. Finally, we analyzed the expression of RASSF10. Elevated RP11-63A11.1 significantly increased the expression of RASSF10 (Additional file 6: Fig.S3). Thus, we believe that introduction of RP11-63A11.1, as a ceRNA, elevated RASSF10, inducing apoptosis in pRCC cells.

**Table 3:** Subcellular location of RP11-63A11.1

Subcellular locations	Score
Cytoplasm	0.545466739277
Nucleus	0.362290233319
Ribosome	0.0218256001327
Cytosol	0.0265381009603
Exosome	0.0438793263113
Predicted location	Cytoplasm

## Discussion

Similar to ccRCC, pRCC patients are usually diagnosed at an advanced stage or metastasis. The major treatment for local pRCC is surgical operation. However, approximately 40% patients reoccur after surgical resection, resulting in a worse prognosis [25]. In recent years, increasing researches of cancers focused on molecular characteristic in early diagnosis and prognosis improvement. Of note, lncRNAs have been revealed to function as main regulators in various biological processes [26, 27]. Notably, it was believed that cancer-related lncRNAs may be helpful for researchers to understand the mechanism of cancer progression and develop efficient measures to improve the prognosis [28]. And cancer-related lncRNAs have been described in some cancer types, such as esophageal adenocarcinoma [29] and prostate cancer [30]. In the present study, we obtained 1872 caner-related lncRNAs through integrating Immlnc and TCGA databases and screened 367 significant DECRLs in pRCC. Cox regression analysis suggested that 26 significant DECRLs were related to the survival of patients with pRCC, among which 10 lncRNAs (RP11-573D15.8, LINC01317, RNF144A-AS1, TFAP2A-AS1, LINC00702, GAS6-AS1, RP11-400K9.4, LUCAT1, RP11-63A11.1, RP11-156L14.1) were enrolled into the risk model to generate a cancer-related lncRNA signature in pRCC for survival prediction. More surprisingly, the signature was a prognostic risk factor from other clinical features in pRCC.

Based on this signature, survival curves suggested that patients in high risk group had a poorer survival rate than patients in low risk group. Additionally, ROC curves exhibited that the precision of this signature was more than 75% for survival prediction at different years. These results were validated in testing set and combination set and similar outcomes were observed, suggesting the signature was a forceful tool for predicting prognosis. What’s more, this cancer-related lncRNA signature was also a forceful tool for prognosis prediction in different classes of some clinicopathological features, including age, gender, and tumor stage.

Other prognostic signatures in pRCC were previously reported. For instance, Wang et al. [31] established a prognostic signature with 15 immune-related genes. Although prognosis of patients could be predicted by their signature, the accuracy of their prognostic signature in predicting prognosis in 1, 3 and 5 years was lower than that of ours (Table 4). In addition, Gao et al. [32] also grouped pRCC patients into training set and testing set. A five mRNAs signature was identified in training set and the accuracy of their signature was 0.82, which was lower than the accuracy in our training set. Although the result of testing set showed that patients could be divided into high and low risk groups by the five mRNAs signature, the accuracy of their signature in testing set was not assessed. Zhang et al. [33] constructed a risk model by 17 mutant genes. The AUC of their signature was 0.907 in 3 years, which was a considerable result. However, the result was not validated in their study and it was unknown whether the mutant-gene signature was efficient for predicting prognosis in 5 years. Similarly, a signature of four lncRNAs was constructed to predict prognosis [34]. In spite of significant accuracy of this signature displayed, a validation result was not provided in their study. Taken together, our cancer-related lncRNA signature might be more beneficial than the previous signatures mainly because of higher accuracy and validation of the findings.

**Table 4:** Comparison of the accuracy of the prognostic signatures from Wang et al. and ours in 1, 3 and 5 years

Sample set	Prognostic signature of Wang et al.			Prognostic signature of this study		
	1 year	3 years	5years	1 year	3 years	5years
Training set	0.934	0.796	0.662	0.967	0.933	0.839
Testing set	0.756	0.695	0.714	0.901	0.788	0.884
Combination set	0.880	0.766	0.678	0.948	0.849	0.869

Among the lncRNAs in our cancer-related signature, RNF144A-AS1, now called GRASLND, was reported to serve as an important regulator in stem cell chondrogenesis [35]. RNF144A-AS1 could also facilitate the migration and invasion of bladder cancer cells [36]. LINC00702 was a newly identified lncRNA and was involved in the progression of several malignancies via tumorigenesis-associated pathways such as Wnt/β-catenin pathway and PTEN/PI3K-AKT pathway [37–40]. LUCAT1 was the only lncRNA widely investigated in human cancers among the ten lncRNAs. LUCAT1 could promote tumorigenesis and development in various cancers. Besides, it could promote anti-tumor drug resistance in some tumor types such as NSCLC and osteosarcoma [41, 42]. These results suggest that these lncRNAs are reasonable and of importance in human cancers. However, to our knowledge, they were firstly uncovered to be novel prognostic biomarkers in pRCC in this study. In consistent with previous researches [17, 18] in which RP11-63A11.1 was reported, we found that RP11-63A11.1 correlated with clinicopathological features and prognosis of pRCC patients. However, its role in pRCC cells was incompletely understood. We revealed that RP11-63A11.1 was decreased in pRCC cells. Furthermore, increased RP11-63A11.1 inhibited the proliferation and induced apoptosis of pRCC cells, indicating RP11-63A11.1 served as a tumor suppressor in pRCC. Subcellular localization of lncRNA transcripts is of great importance when we investigate its mechanism. Therefore, the subcellular location of the RP11-63A11.1 transcript was predicted using lncLocator based on its sequence acquired from LNCipedia. RP11-63A11.1 was likely to be located in the cytoplasm. LncRNA located in the cytoplasm or cytosol probably serves as a ceRNA, and regulates mRNA stability or translation. Regarding that elevated RP11-63A11.1 significantly increased the expression of RASSF10, we hypothesized that RP11-63A11.1 acted as ceRNA, and regulated the stability or translation of RASSF10. Further studies are indicated to investigate more specific mechanisms of RP11-63A11.1 in pRCC.

The roles of other lncRNAs including RP11-573D15.8, LINC01317, RP11-400K9.4, and RP11-156L14.1 were not reported in previous literatures. These lncRNAs were firstly reported in the present study. When combined with the above-mentioned lncRNAs, these lncRNAs generate a powerful tool for survival prediction in pRCC. To explore the biological functions of the cancer-related lncRNA signature in pRCC, we performed WGCNA to seek gene modules associated with this signature. Two gene modules were finally identified. Gene enrichment analysis showed genes in the blue module (positively correlated) were mainly enriched in biologic processes of cell division, proliferation and cell cycle, indicating that pRCC with high risk score has stronger proliferation capability than pRCC with low risk score.

## Conclusions

In summary, we successfully figured out a novel cancer-related lncRNA signature with powerful predictive function for pRCC prognosis. These results of this study may facilitate understanding of molecular mechanisms in pRCC development, and the cancer-related lncRNA signature may become a promising biomarker and therapeutic target in pRCC. Of note, RP11-63A11.1 may serve as a ceRNA and regulate RASSF10, facilitating the understanding of the development in pRCC.

## Supplementary Information

**Additional file 1: Table S1.** The cancer-related lncRNAs with expression data in pRCC.

**Additional file 2: Figure S1.** Risk modes of every LncRNA in the signature. Among all the 10 lncRNAs in the signature, RP11-63A11.1 was the most significant one with the lowest P value (P = 0.0014).

**Additional file 3: Figure S2.** RP11-63A11.1 was introduced into SK-RC-39 and A498 cells. After introduced into SK-RC-39 and A498 cells, two clones in each cell line were generated and the level of RP11-63A11.1 was significantly increased.

**Additional file 4: Table S2.** Sequence of RP11-63A11.1 obtained from LNCipedia.

**Additional file 5: Table S3.** The genes which are positively correlated with RP11-63A11.1

**Additional file 6: Figure S3.** Elevated RP11-63A11.1 significantly increased the expression of RASSF10.

## Data Availability

All data generated or analyzed during this study are included in Additional files.

## Abbreviations

pRCC: Papillary renal cell carcinoma
lncRNA: Long non-coding RNA
TCGA: The Cancer Genome Atlas
DECRLs: Differentially expressed cancer-related lncRNAs
ROC: Receiver operating characteristic
OS: Overall survival
AUC: Area under curve
PFS: Progression-free survival
DSS: Disease-specific survival
GO: Gene oncology
ceRNA: Competing endogenous RNA
DEMs: Differentially expressed mRNAs
