# Supplementary figures and images for: A novel prognostic cancer-related lncRNA signature in papillary renal cell carcinoma
Source: Cancer Cell Int. 2021 Oct 18;21:545. doi: 10.1186/s12935-021-02247-6 (PMC8525017; doi:10.1186/s12935-021-02247-6)

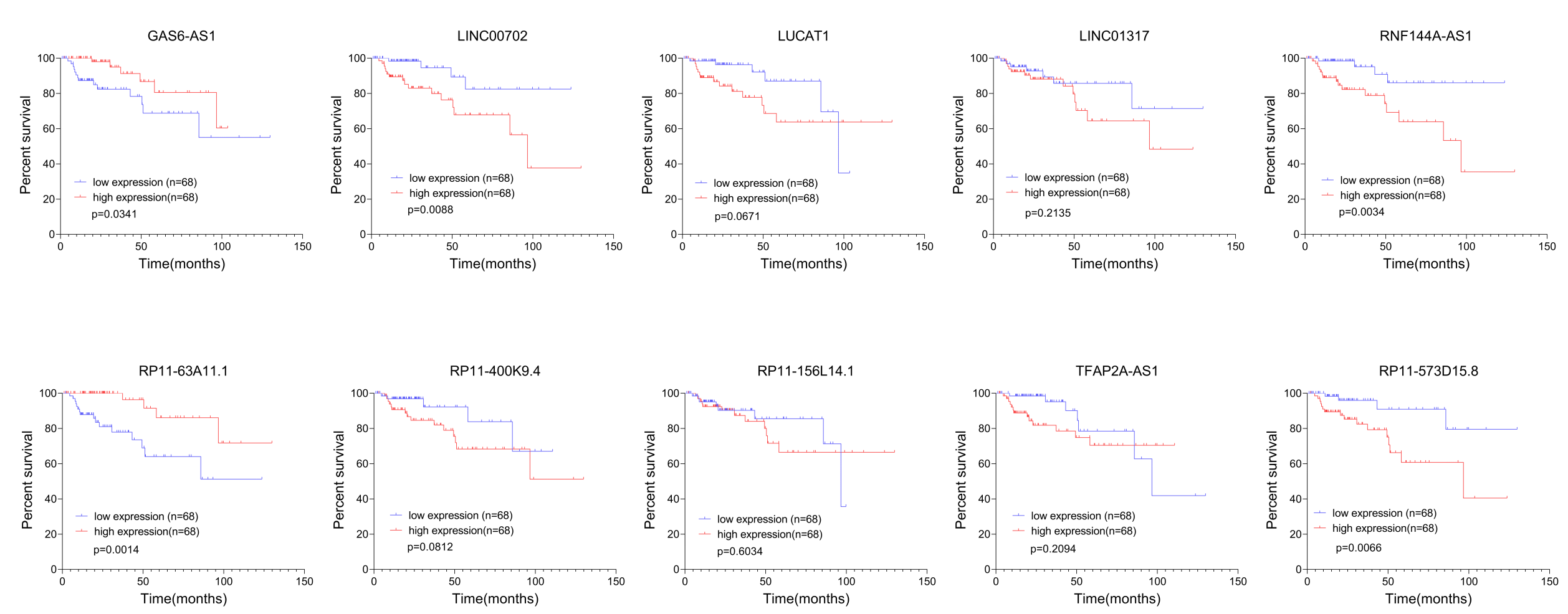

Supplement: Supplementary file 2 — Additional file 2: Figure S1. Risk modes of every LncRNA in the signature. Among all the 10 lncRNAs in the signature, RP11-63A11.1 was the most significant one with the lowest P value (P = 0.0014). [file 12935_2021_2247_MOESM2_ESM.tif]

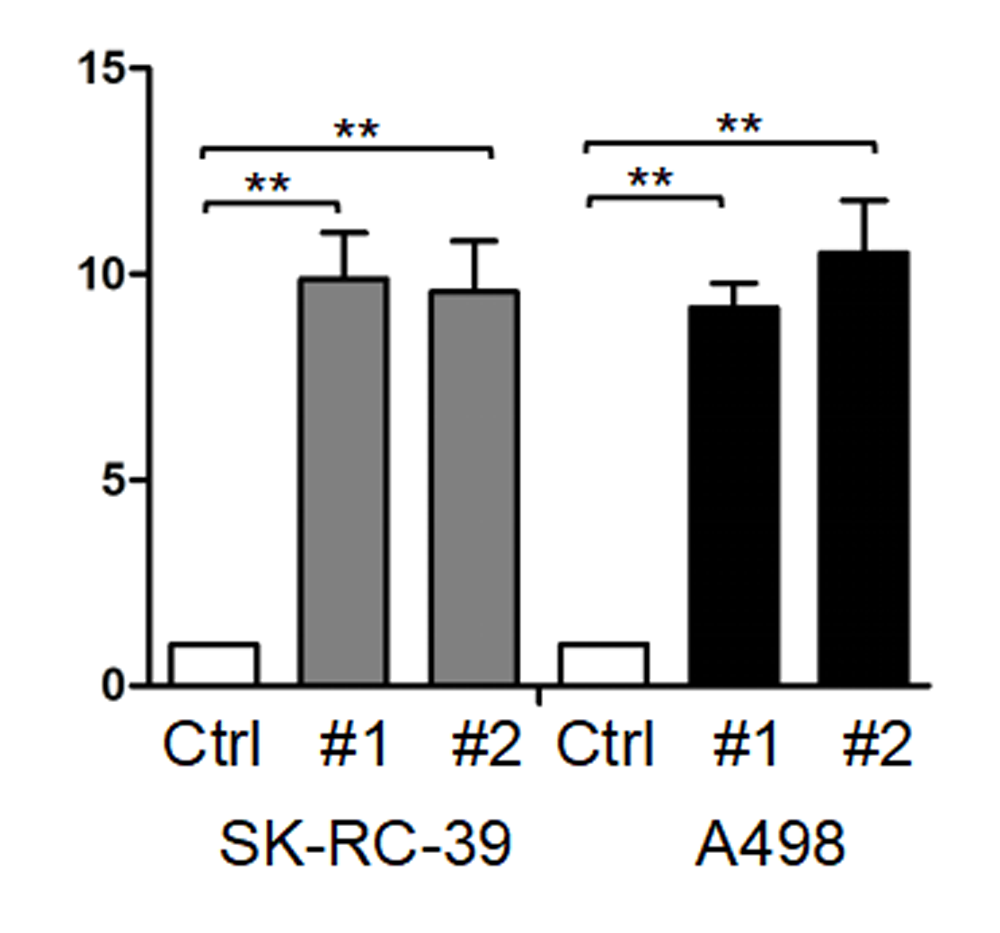

Supplement: Supplementary file 3 — Additional file 3: Figure S2. RP11-63A11.1 was introduced into SK-RC-39 and A498 cells. After introduced into SK-RC-39 and A498 cells, two clones in each cell line were generated and the level of RP11-63A11.1 was significantly increased. [file 12935_2021_2247_MOESM3_ESM.tif]

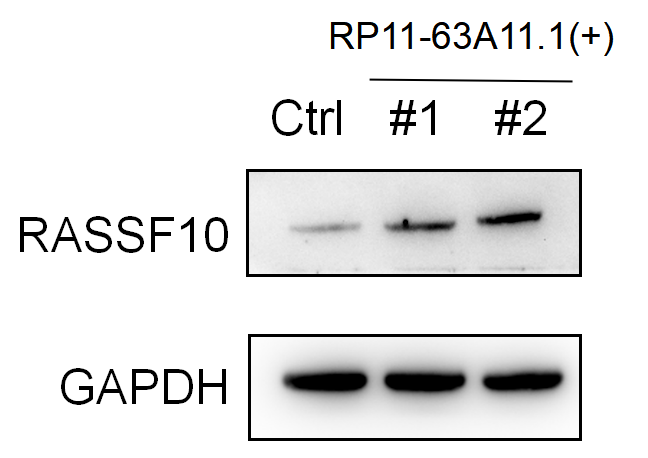

Supplement: Supplementary file 6 — Additional file 6: Figure S3. Elevated RP11-63A11.1 significantly increased the expression of RASSF10. [file 12935_2021_2247_MOESM6_ESM.tif]
