# Supplementary material for: A novel prognostic cancer-related lncRNA signature in papillary renal cell carcinoma
Source: Cancer Cell Int. 2021 Oct 18;21:545. doi: 10.1186/s12935-021-02247-6 (PMC8525017; doi:10.1186/s12935-021-02247-6)
Supplement: Supplementary file 4 — Additional file 4: Table S2. Sequence of RP11-63A11.1 obtained from LNCipedia. [file 12935_2021_2247_MOESM4_ESM.docx]

Table S2. Sequence of RP11-63A11.1 from LNCipedia.

| AGAGAGGCCAGCCCCCATTCCTGGGGCTCCCCGAGGAGCTGGCTTTCAGCAGGGTTTAGAGCAGCACGAAAAGTGAACTGACCCTGGAAAAATGGTAATCTTCTCATCTGTGAGGATATGGAACCCCAACCTCTTCCTGGACACCTGATGATCTGCTTGTGATGGGCTCAGAGTCTTGAAACACAGAACTATGAGCTCATCTCATATCCCAATCCAGCAGCATGGAAACCTCAGACTGCAAGGCCCAAGACTGGCACTTGTTCTCTCCCAACTCTTTTCTTTCTCTCTCTCCTTTCTTTTATCCCTTAATTCCTTCTTGCTTCCTTCCAAGATTTATACTATTACCTTTTAGGCAAAACATCCTGAACATGTAAAATAAACTAATTAAAATCAA |
| --- |
